# Possible Sources of Bias in Primary Care Electronic Health Record Data Use and Reuse

**DOI:** 10.2196/jmir.9134

**Published:** 2018-05-29

**Authors:** Robert A Verheij, Vasa Curcin, Brendan C Delaney, Mark M McGilchrist

**Affiliations:** ^1^ Netherlands Institute for Health Services Research Utrecht Netherlands; ^2^ King's College London London United Kingdom; ^3^ Imperial College London Imperial College Business School London United Kingdom; ^4^ University of Dundee Department of Public Health Sciences Dundee United Kingdom

**Keywords:** electronic health record, data accuracy, data sharing, health information interoperability, health care systems, health information systems, medical informatics

## Abstract

**Background:**

Enormous amounts of data are recorded routinely in health care as part of the care process, primarily for managing individual patient care. There are significant opportunities to use these data for other purposes, many of which would contribute to establishing a learning health system. This is particularly true for data recorded in primary care settings, as in many countries, these are the first place patients turn to for most health problems.

**Objective:**

In this paper, we discuss whether data that are recorded routinely as part of the health care process in primary care are actually fit to use for other purposes such as research and quality of health care indicators, how the original purpose may affect the extent to which the data are fit for another purpose, and the mechanisms behind these effects. In doing so, we want to identify possible sources of bias that are relevant for the use and reuse of these type of data.

**Methods:**

This paper is based on the authors’ experience as users of electronic health records data, as general practitioners, health informatics experts, and health services researchers. It is a product of the discussions they had during the Translational Research and Patient Safety in Europe (TRANSFoRm) project, which was funded by the European Commission and sought to develop, pilot, and evaluate a core information architecture for the learning health system in Europe, based on primary care electronic health records.

**Results:**

We first describe the different stages in the processing of electronic health record data, as well as the different purposes for which these data are used. Given the different data processing steps and purposes, we then discuss the possible mechanisms for each individual data processing step that can generate biased outcomes. We identified 13 possible sources of bias. Four of them are related to the organization of a health care system, whereas some are of a more technical nature.

**Conclusions:**

There are a substantial number of possible sources of bias; very little is known about the size and direction of their impact. However, anyone that uses or reuses data that were recorded as part of the health care process (such as researchers and clinicians) should be aware of the associated data collection process and environmental influences that can affect the quality of the data. Our stepwise, actor- and purpose-oriented approach may help to identify these possible sources of bias. Unless data quality issues are better understood and unless adequate controls are embedded throughout the data lifecycle, data-driven health care will not live up to its expectations. We need a data quality research agenda to devise the appropriate instruments needed to assess the magnitude of each of the possible sources of bias, and then start measuring their impact. The possible sources of bias described in this paper serve as a starting point for this research agenda.

## Introduction

### Electronic Health Records: A Potential Goldmine

Researchers have long seen the reuse of large-scale, routine health care data as a means of efficiently addressing many research questions of interest. In the United Kingdom, there has been almost 25 years of research using routine primary care data, anonymized at source, through the General Practice Research Database (now CPRD, Clinical Practice Research Datalink [1]), and other data sources, also pooling data from multiple practices and tied to specific electronic health record (EHR) systems (QResearch [2], ResearchOne [3]). A similar development has taken place in the Netherlands, where, in the early 1990s, the Netherlands Institute for Health Services Research (NIVEL) developed its Netherlands Information Network of General Practice [4], now named NIVEL Primary Care Database (NIVEL-PCD) [5,6]. Belgium also has its Intego Network [6,7] and France, until recently, had its l’Observatoire de la médecine générale société [8]. These databases provide valuable information about the use of health services and developments in population health. In the United States, there has not been a tradition of using routine anonymized data, largely because the Health Insurance Portability and Accountability Act (HIPAA) regulations place restrictions on the linkage of health data from different sources without consent [9-11] and because small office practices have not been widely computerized. Instead, the focus has been mainly on secondary care (hospital) data, facilitated by the National Institute of Health’s (NIH) Clinical Translational Science Awards (CTSA) [12]. Use or reuse of administrative data for research purposes is becoming more restricted in Europe as well, partly as a consequence of the European General Data Protection Regulation (GDPR) that was established in 2016 [13,14]. In addition, data owners increasingly want control over the use of their data, making it more difficult to construct large centralized databases.

In recent years, new institutions, networks, and informatics tools have appeared, most of them focusing on secondary care and the development of new treatments. For example, the i2b2 platform has proven popular as a means of structuring clinical data, with tools for distributed querying [15]. Networking between the CTSA sites and additional access to primary care health record data have been promoted by the Patient Centered Outcomes Research Institute (PCORI) and its PCORnet distributed data network [11,16] and the US Food and Drug Administration’s sentinel database [17].

As more data have become available, so has the funding for research projects to utilize it, such as the Big Data to Knowledge initiative in the United States [18], and the IMI European Medical Informatics Framework [19]. The recently established European institute for Innovation through Health Data (i~HD [20]) also promotes extensive use or reuse of health care data. Increasingly, EHR data are staying where they are, queries are being run across multiple datasets, and large-scale analytics techniques such as data mining or machine learning are being used.

### Learning Health Systems and Data Quality

These developments provide a foundation for using routine EHRs in support of a “learning health system” (LHS) [21,22]. An LHS is a system in which knowledge generation and reapplication is a natural product of the health care delivery process and leads to continuous improvement in outcomes and institutional performance [23]. In such a system, routine health data are analyzed and fed back to the health care providers and patients that provided the data, using reports, decision support systems (DSSs), or any other type of feedback method. These data are also used or reused for research that is relevant for clinical practice and/or health policy.

However, it is widely recognized that data collected for one purpose may not be suitable for another and that there are serious issues to be considered in the use or reuse of EHR data [24-28]. There are some strong opinions that data shall be used only for the purpose for which they were collected and that data should not be used if a purpose was not defined before the collection of data [29]. An alternative view, formulated by Juran [30] in 1954 (and reformulated in 2006 by De Lusignan et al [31]), is that: “data are of high quality if they are fit for their intended uses in operations, decision making and planning.”

It is this latter definition of data quality that enables the possibility of data use or reuse. Juran’s statement is also a warning against the view that sufficiently large and diverse amounts of data will allow us to disregard the quality and provenance of data. More data do not substitute for fit data and fit cannot be judged without knowing the purpose for which the data are to be used. Even inaccurate data can be useful data if the purpose is, for example, to study the quality of data being used by health professionals. Understanding the mechanisms behind variations in data quality is particularly important in the “Big Data” era and for further pursuing the principles of an LHS. The principal aim of this paper was to create awareness among potential and current users of primary care EHR data of the factors that influence the quality of these data and to open the discussion regarding what can be done to deal with these factors. In doing so, we address the following questions:

1. How do EHR data flow from their original source to any form of use or reuse?
2. What are the purposes for which EHR data are used or reused?
3. To what extent may different purposes and the nature of the data flow constitute possible sources of bias?

In this discussion paper, we first describe the steps or stages involved in collecting and processing EHR data. This is followed by a description of the purposes for which the data are and can be used. And finally—given the purposes and the data collection steps—we identify a number of possible sources of bias involved in the use or reuse of EHR data.

## Methods

First, this study is based on the author’s discussions during the Translational Research and Patient Safety in Europe (TRANSFoRm) project [32]. The European Commission FP7 sponsored project TRANSFoRm 2010-15 sought to develop, pilot, and evaluate a core information architecture for the LHS in Europe. Second, it is based on the authors’ extensive experience in using and reusing EHR data for research (all authors), as cofounder of one of the largest primary care databases in Europe, NIVEL Primary Care Database (RV), as health informatics experts (MM and VC), as well as on their experience as a practicing general practitioner (BD).

One of the objectives of the TRANSFoRm project was to develop tools to assess the quality of EHR data for secondary use. We first assessed the flow of data involved in basically any use or reuse of EHR data, using the privacy and confidentiality framework developed in the project [33], involving the flow of data from a care zone to a database zone, to a research zone, then assessed the different purposes for which these data are and can be used, and finally, we mapped possible sources of bias associated with each of the purposes onto the stages involved in data collection and processing.

## Results

### Data Flow

In general, data flow from their initial point of generation through one or more systems for processing, ultimately generating information for a desired purpose and creating opportunities for reuse. At any stage in the flow, the data can be wholly characterized in terms of completeness, correctness, and precision relative to purpose.

In terms of the TRANSFoRm Zone Model described by Kuchinke et al [33,34], data move from the care zone to the research zone. The care zone is where health care professionals provide care to their patients, “the area of patient diagnosis and treatment.” It is where “personal data are stored and used within the care context by the treating physician.” The noncare zone contains “research databases and secondary use databases that have been derived from primary medical care data.” In the research zone, “the researcher receives data suitable for processing and analysis in specific research projects, addressing specific research questions […].” [34].

**Figure 1:**
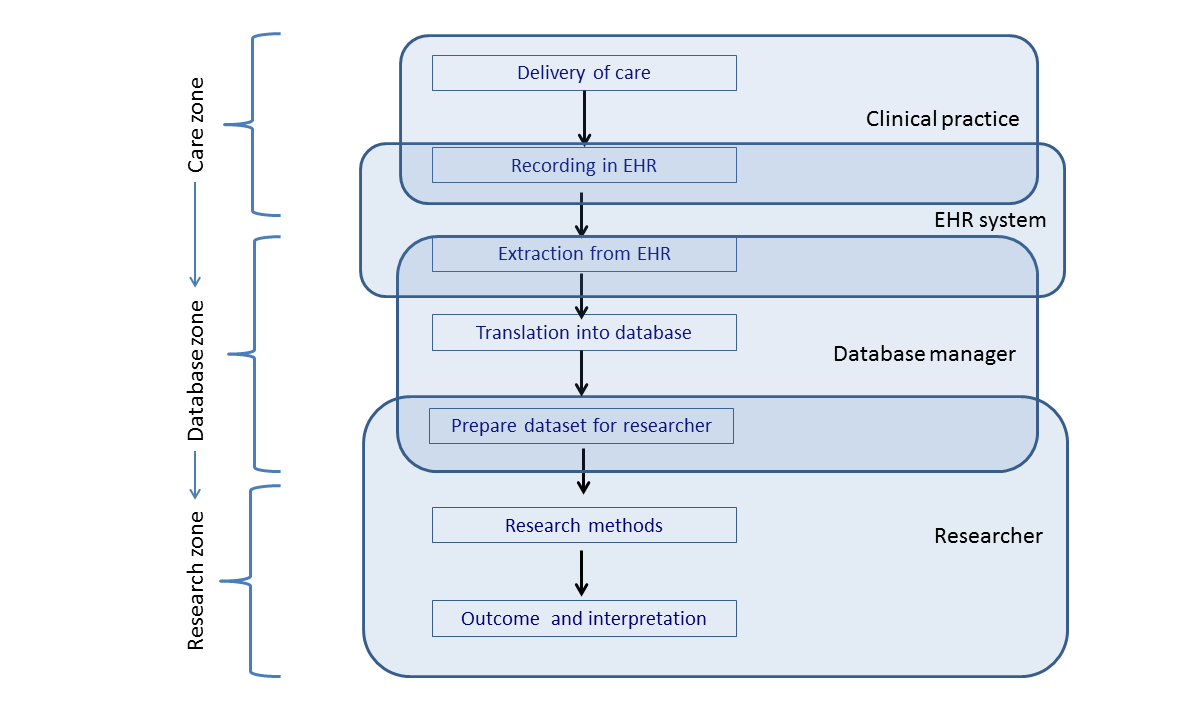
Steps and actors involved in the data flow between the delivery of care and applications reusing the data. EHR: electronic health record.

The TRANSFoRm Zone Model was extended with a number of substeps or stages within each of the zones and by naming the different actors involved in each step: health care providers, EHR vendors, data stewards, and researcher/analyst. These stages and the principal actors involved in each of them are depicted in Figure 1.

To avoid redundancy, the distinct stages will be discussed in more detail in the “sources of bias” section.

### Purposes

EHRs data can be used and reused for many purposes. An extensive overview is provided by Safran et al [35]. Here, we distinguish 3 broad categories: managing individual patient’s care (including also DSSs), management of organizations (including performance indicators), and various types of medical and health services research.

#### Care for Patients

Electronic health data are primarily recorded to document and facilitate the care for an individual patient. However, many patients receive health care from a variety of health care providers, and sharing relevant information among these health care providers on patients’ health problems and treatments is becoming increasingly important. There is an increasing exchange of information between primary care physicians and their nurses within a practice, between primary care and hospital care, pharmacies, out-of-hours services, etc. In the Netherlands, this gave rise to the “national switchboard” initiative that allows health care professionals to see “professional summaries” of a patient’s medical history. This project was subsequently voted down in Parliament, but restarted in 2015 [36]. In the United Kingdom, the NHS National Programme for IT that was to provide a centrally held summary care record (termed “care.data” more recently) was also terminated [37]. In the United Kingdom, summary data for major diagnoses, allergies, test results, and medications are shared nationally, and locality schemes exist for sharing “views” of records between primary care and hospital sites. However, patient access is regarded as a means to empower patients and enhance self-management, and remains high on the political agenda, at least in the Netherlands and the United Kingdom.

To enable useful sharing of EHR data between professionals and patients, the data should be complete, correct, and precise, relative to health care needs. As more use is made of health data, the more serious the consequences of incomplete, incorrect, or imprecise data, particularly in relation to comorbidity, comedication, allergies, and other intolerances.

EHR data are also increasingly used to enable DSSs [38-41]. For example, almost all Dutch general practitioners (GPs) use an evidence-based electronic prescribing system [42]. EHR data can be used to generate algorithms for DSS and also as a source of data in clinical practice. In either case, a DSS requires stringent data quality to function correctly, especially with respect to diagnosis and prescribing medication.

#### Management Information

EHR data are also increasingly used to calculate quality-of-care indicators for managers within the health care facility itself, or as a source of information for third-party organizations such as health insurers or governmental bodies. This can be problematic [43]. For example, in the Netherlands, Stirbu-Wagner et al found, in 2008, that it was difficult to retrieve the necessary data from EHR systems. Technically, the data elements could be extracted from the EHR systems, but the quality of the data, in relation to the required purpose, was poor. Similar results were found in more recent Dutch studies [44,45]. However, the situation regarding EHR data quality within primary care in the Netherlands is likely to have changed in recent years. Substantial numbers of practitioners (>90% of the Dutch GPs in 2013 [46]) receive feedback on the quality of their recording, based on the data quality feedback tool developed by NIVEL, as well as the fact that a portion of the reimbursement of GPs was based on the quality of recording [46,47]. Similarly, in the United Kingdom, the Quality and Outcomes Framework (QOF) promoted completeness of recording for agreed data elements within the EHR. These examples suggest that higher-quality data become more available if reimbursement is dependent on it [10]. It also illustrates how the reimbursement system can affect data quality, particularly in regard to systematic distortion of disease prevalence on the basis of the codes entered (eg, coding depression as “low mood” rather than “depression”) [48].

#### Research

Increasingly, EHR data are also used in observational studies, recruitment and follow-up in clinical trials, and health services research. Although there are also distinct disadvantages (one of which is uncertainty about the quality of data; the subject of this paper), in comparison with surveys, EHR data for scientific research have several important advantages, suffering less from systematic errors such as selective nonresponse, response bias (systematic error caused by social desirability or leading questions), and recall bias (systematic error caused by differences in the precision or completeness of the recollections of events or experiences from the past). Moreover, EHR data are generally recorded continuously and routinely rather than periodically.

EHR systems serve as a source of data for monitoring the health of populations, allowing researchers to evaluate, among others, the effects of environmental hazards [49]; the impact of health system reforms [50,51]; how health care systems function; and developments in public health, all at comparatively low cost. In addition, linking these EHR data to other distinct data sources increases the research possibilities enormously. For example, data from NIVEL’s Primary Care Database [5] have been linked to many other data sources providing environmental characteristics [52,53], migration background [54], income, school dropout rates [55], insurance claims [56], and pharmacy data [57]. EHR data are also increasingly used for public health forecasts and surveillance [58,35,59,60]. The research potential of EHR data is also increasingly recognized outside the Western world [61].

EHR data have a distinct advantage over claims data as they are generated as part of the health care process and can potentially be extracted in real time, whereas claims data usually only become available after the treatment and claims processes have been completed. Depending on the health care system, this can take months or even years. The added value of hospital EHR data over claims data was clearly illustrated by Amarasinham [56]. In addition, primary care data have the advantage of containing data from before (and after) hospitalization.

More recently, routine EHRs are increasingly seen as a viable source of data for clinical trials [24,62]. EHR data constitutes a large part of what is called real-world data. By most definitions, real-world data are data that are collected in a usual clinical setting, as opposed to a research clinic [63]. EHR data are increasingly used alongside registry data and patient-recorded data (see for example [64]), all of which can provide contextual information that enriches the data collected directly in controlled trials. Such use of routinely recorded data in the so-called real-world studies aims to address the efficacy-effectiveness gap in drug trials, where a drug performs worse in a real-life context when compared with a trial. Furthermore, EHR data can be used to assess the feasibility of trial criteria and to target sites for recruitment that have relatively high numbers of eligible patients.

### Sources of Bias in the Electronic Health Records Data Chain

There are a number of reasons why data may not be fit for a given purpose. To review these reasons, we describe the series of steps that lead from a clinically relevant event that takes place in a health care setting to an application reusing the data. These steps can be regarded as a data food chain. Analogous to a real food chain, any contamination, or “bias” in any of the steps will have consequences for the remaining steps. For each of the steps or stages, the factors that may affect data quality are described below.

#### Step 1: Delivery of Care (There Must Be an Event That Can Be Recorded)

This step may seem trivial, but (eg) for a blood pressure (BP) reading to be recorded, the measurement must first take place. The actors involved in this step are a health care professional interacting with a patient. The likelihood of such a measurement to take place is partly dependent on factors related to the health care system. Obviously, whether a BP measurement takes place is of course primarily dependent on the GPs professional judgment in relation to this individual patient. BP may be clinically relevant or necessary to reassure the patient. However, this judgment is dependent on a number of other factors, most of which are strictly medical and related to that individual patient, but there are a number of other factors that may systematically affect the decision to measure a patient’s BP as well. For example, as explained below, there are different incentives in the United Kingdom and the Netherlands to record BP. This difference will result in almost complete recordings for the whole population in the United Kingdom, whereas in the Netherlands, there will only be complete recordings for people known to have a chronic disease such as diabetes for which BP readings are relevant. These factors need to be known to anyone using the data in any of the subsequent steps.

First, organizational aspects of the health care system will affect actual medical practice and thereby the opportunity for an event to be recorded. For example, the difference between gatekeeping systems and nongatekeeping systems determines the population, and thereby the denominator, in epidemiological studies. In gate-keeping systems, patients need a referral from a GP before being able to make an appointment with a medical specialist, and usually GPs have a more or less stable patient list [65]. In terms of data quality, such gate-keeping systems have one very important advantage, because they allow for the calculation of an epidemiological denominator. Ideally, prevalence and incidence are expressed per 1000 in the population. This population must therefore be known. Nongatekeeping systems have only the consulting population to report on, whereas in gatekeeping systems, GPs have a more fixed list of patients that can be followed through time [7].

Gatekeeping affects the numerators as well. For example, in a nongatekeeping system, a BP reading may take place outside primary care, resulting in fewer BP readings in primary care settings. Similarly, the existence of a list system, where people are listed as members of the practice population, may not affect the number of BP readings in primary care as a whole, but it will affect the number of BP readings by a particular doctor. Health care system differences such as these have been found to be responsible for international differences in prevalence and incidence of chronic diseases [66,67].

Second, the reimbursement system in one country may stimulate BP readings under certain circumstances, whereas in other countries, it will not. In the Netherlands, prevailing quality of care indicators require BP readings to be scheduled to take place every year for patients with chronic diseases such as diabetes and cardiovascular problems. This is incorporated in the pay for performance part of the GP reimbursement system for these patients in the Netherlands but only for these patient groups. In the United Kingdom on the other hand, the QOF promotes BP readings for the whole population each year [31]. It should be noted that incentives within the health care system may seem to affect completeness of the data, but in this example, it merely reflects differences in medical practice that create data-recording opportunities.

Third, professional guidelines vary across health care systems. If a professional guideline says a BP reading should be done every year in a certain population, it will be more likely that such a measurement takes place (and get recorded).

Fourth, high practice workload may have a negative effect on taking regular BP measurements.

These 4 factors determine whether any intervention takes place in clinical practice, thereby creating a data-recording opportunity. Analysts using data from different health care systems should be aware of these factors. In any of the subsequent steps, differences in data-recording opportunities may be perceived as differences in data quality, but they are not, as they reflect real differences in medical practice. Averaging BP recordings in the United Kingdom and in the Netherlands, using the whole population as the denominator, will render invalid results because the health care system promotes readings in a much larger patient population in the United Kingdom as compared with the Netherlands, where distinct populations of chronically ill patients are targeted.

#### Step 2: Recording in Electronic Health Record (An Event That Is Not Recorded Will Not Be Present in Any Dataset)

There are 2 actors involved in this step: the health care professional that does the recording and the EHR vendor’s software. Whether an event gets recorded is dependent on several factors.

First, there must be a software system actively used by the health care professional. About 99% of practices in the United Kingdom and the Netherlands are today using an EHR system, but this is not the case in the United States and many other countries. In general, functionalities available within the EHR systems may affect the completeness, correctness, and precision of recorded data. Although all software packages in the Netherlands and in the United Kingdom are certified by their respective authorities, considerable differences between packages have been reported in terms of what is actually recorded. For example, considerable differences between primary care EHR software brands were found in the recording of contraindications, episodes of care [68,69], as well as in the quality of prescribing [70]. The most probable factor here is the design and user interface of the software packages involved, but little is known about the actual mechanisms behind these differences. Perhaps, the holistic framework proposed by Van Gemert-Peijnen et al may prove to be useful here [71].

Second, health care professionals may display strategic recording behavior, for example, as a result of monetary incentives. Enhanced reimbursement schemes for chronically ill patients will encourage GPs to diagnose patients with chronic disease. Upcoding has been found to be a risk in relation to diagnosis-related groups used as a basis for reimbursement [72]. In addition, monetary incentives may lead to selective recording habits. For example, Mukherjee et al found that the QOF affected the recording of allergies [73]. This type of strategic behavior may lead to incomplete and incorrect data or both, as incorrectness usually implies incompleteness as well. Moreover, the fact that there are companies providing services to health care facilities to “optimize” their cash flows suggests that there are incentives for strategic recording behavior. As we know that part of the cash flow is dependent on EHR data, it is likely that strategic recording behavior can have an effect on the quality of the data, especially in systems where billing codes and reimbursement fees are related to recorded diagnoses (as is the case in many countries).

In the United States more than in the EU, health care facilities can get involved in lawsuits with high financial risks. This can result in another form of strategic behavior related to the health care system and lead to differences in quality of the data being recorded either in a positive or negative way.

In addition, awareness of sharing data with other health professionals or patients may have an effect on whether an event gets recorded, and on the way it gets recorded. For example, health care professionals may be more reluctant to record an uncertain diagnosis in situations where this information is shared with colleagues. The size of this effect will be dependent on characteristics of the event involved, on the health professional concerned, and on whether he/she is of the same profession and/or in the same health service organization. A health professional may, for example, be more hesitant to record depression as a diagnosis than diabetes, and this may vary substantially between health professionals. Similarly, GPs may be more hesitant to record a patient’s excessive alcohol intake if this information is shared with other professionals. GPs may be less hesitant to share information with GPs than with medical specialists or mental health services.

By facilitating patients' access to EHRs, patient empowerment is part of health policy in many countries [74]. Although very few patients have used this capability thus far, there may be serious consequences in terms of selective or biased recording of information. Quite paradoxically “enforced” sharing of data may lead to incomplete, incorrect, or imprecise data.

Recording behavior will also be dependent on the existence of recording guidelines. In some health care systems, there may be guidelines describing what should be recorded in an EHR system and when [75-77]. In other countries, such guidelines may not exist. Absence of recording guidelines may lead to less precise, less complete, and less correct data.

The available coding systems and thesauruses built into EHR systems determine what will and can be recorded. For example, in the International Classification of Primary Care [78], there are only about 600 codes for diagnoses and symptoms, whereas coding and classification systems such as Read, the Systematised Nomenclature of Medicine, or the various versions of the International Classification of Diseases have many more codes of greater semantic complexity and may prove more difficult to use in primary care settings, resulting in inconsistent recording.

Two other factors at the level of health care professionals will affect adequate use of EHR systems: knowledge and time. Software packages and coding systems may enable health care professionals to do all that is required and recording guidelines may tell them what to do, but if health care professionals are not familiar with these systems and guidelines, there will still be sub-optimal use of the EHR system, leading to incomplete or incorrect data and use of free text where it is not necessary. Parsons et al [79] report a “profound” data quality improvement after providing training and documentation to primary care services in New York. The effect of feedback on data quality is reported by Van der Bij et al [80]. This feedback makes practitioners aware of the importance of high-quality recording and of the differences among them.

Moreover, the health care professional’s workload may play a role. Shortage of time in a consultation will not stimulate proper recording behavior.

Lack of knowledge and time will inhibit appropriate use of the EHR systems and lead to extensive use of free text or no recording at all. The use of free text is generally regarded as problematic and only useful for small-scale studies, unless this free text can be turned into data that can be processed automatically [81]. Within the international context, this difficulty is magnified by the presence of many languages and target coding systems with national variations and varying accuracy. DSSs have an important secondary role in supporting data quality in the EHR if their operation results in more codes being placed in the EHR [82].

#### Step 3: Extraction From Electronic Health Record (Data Must Be Extracted for Further Analysis or Reporting)

Unless data are only used within the recording practice (the care zone, in terms of the TRANSFoRm Zone Model [34]), it needs to be extracted and transported to another site.

The actors involved in this step include the health care professional in a governance role, the software vendors who are responsible for the necessary software components (receiver as well as sender), and patients.

The database experts together with the software vendors are responsible for the extraction process from a technical point of view. It is the extraction software and associated queries that determine what data elements are extracted and how this is achieved. Different extraction tools, working in combination with different EHR systems, may render different results [83]. This may lead to incomplete and/or incorrect data. Moreover, extraction tools need to be maintained and adapted to changes in the structure and content of the EHR software. Usually—because detailed knowledge of the structure of the EHR software is needed—it is the software vendor/manufacturer that is responsible for the extraction software. How this extraction software actually works is often not explained as the process is protected by intellectual property rights. Those involved in the subsequent steps can only judge the quality of the extraction tools on the basis of the outcomes, if at all.

The third actor involved in the extraction process is the patient. Privacy regulations may allow patients to object to sharing of “their” data with other health care professionals or for research through an opt-out system, or by not giving consent. Similarly, some practices will allow the use of “their” data and others will not. Data governance options may lead to more or less incomplete or incorrect data for some patients.

#### Step 4: Translation Into Database (Extracted Data Must Be Redatabased as Preparation for Further Analysis or Reporting)

Actors involved in this step include database experts, database staff and domain specialists in the database zone, as the database will be engineered for particular purposes.

First, whether extracted data are actually imported into a database is dependent on the capacity of that database to capture the data that are extracted. This is particularly important in cases where data arrive in multiple formats and coding schemes. These may vary over time, being dependent on, for example, changes in the reimbursement system. The term semantic integration encompasses these issues. When data from different sources are involved, it will almost certainly be necessary to deal with different coding schemes and classifications.

#### Step 5: Prepare Dataset for Researcher (Generating a Research Data File)

Normally, researchers do not do their analyses on the data within the database, but on a dataset that is derived thereof. Not all variables in a database may be relevant or appropriate for a particular study and may be excluded from the research data file. In fact, the “need to know” principle demands that data that are not needed for a particular research question are not transferred to a researcher.

Determining what data are actually needed for a research question is primarily a responsibility of the researcher together with the database manager. These actors have great impact on the content of the dataset that will be analyzed. For example, quality checks or filters may be employed after data are read into the database (step 4). This means that not all data that are in a repository will go into a data file that is used by a researcher for an agreed purpose.

Furthermore, where data are linked, the resulting database may hold only data on the population common to both sources. This will affect completeness of the data. Complete data will only be available from the population that the 2 (or more) linked datasets have in common.

And finally, a repository may not be able to facilitate all types of research. There may be regulations and steering committees that will or will not grant the possibility to use a certain repository for a certain purpose. This will affect the completeness of the extracted data.

#### Step 6: Analysis, Outcomes, and Interpretation

These steps are in the research domain in terms of the TRANSFoRm zone Model. Here, we find the end users of exported EHR data. Different researchers will make different choices with respect to the method of analysis and what they report. Different methods may render different results, even with the same data, as was demonstrated by De Vries et al using data from the General Practice Research Database [84]. Moreover, Reeves and coworkers [50] found that different methods for computing quality-of-care scores can lead to different conclusions. This illustrates that research methods have to be based on knowledge about all previous steps and awareness of each of the possible sources of bias in each step mentioned above.

## Discussion

In the previous sections, we identified 13 possible sources of bias, associated with different steps in the data chain. Textbox 1 summarizes these possible sources of bias that emerge from the combination of purposes and steps in the data chain.

### Awareness and Scope

Awareness of these sources of bias is not self-evident for many that use or reuse EHR data. Where routine electronic health data are readily available, there is a risk of misinterpretation if users are unaware of the different systemic sources of bias and how they interact. It must be emphasized that large volumes of data do not reduce systematic errors, but we do contend that using these data for multiple, distinct purposes is possible, on the condition that users are aware of the risks involved and have strategies for managing them.

This is particularly important when data from different sources and from different countries are being combined in research projects such as the TRANSFoRm [32] project already mentioned, the Electronic Health Record for Clinical Research (EHR4CR) project [85], and the electronic Health Indicator Data (eHID) project [66]. Researchers should be aware of possible sources of bias and take adequate measures to ensure that their research results are not undermined.

This is all the more important because access to data is no longer a privilege of the research community, where individuals are educated and trained to deal with large amounts of data. Academically trained researchers were often the ones that were responsible for the collection of the required data as well as the analyses. Today, this too is no longer the case. Large amounts of data are open and available to the general public, and researchers using the data are very often not the ones who have collected them.

**Possible sources of bias in the use or reuse of electronic health record data that have to be incorporated in the choice of research methods and interpretation of results.**

1. Health care system bias, emanating from:
  - Reimbursement system, pay for performance parameters
  - Role of general practitioner in the health care system; gatekeeping/nongatekeeping
  - Professional clinical guidelines
  - Ease of access by patients to their records
  - Data sharing between health care providers
2. Practice workload
3. Variations between electronic health record (EHR) system functionalities and lay-out
4. Coding systems and thesauruses
5. Knowledge and education regarding the use of EHR systems
6. Data extraction tools
7. Data processing—redatabasing
8. Research dataset preparation
9. Research methodologies

The question then arises: is it possible to provide sufficient metadata to prevent mistakes in using these data? Will the users of these data be able to understand and use this information? Will they be able to allocate enough time for that? Is it possible to set requirements for users of a dataset?

The variation in quality found within any body of data when directed at different purposes may slow down the adoption of an LHS by further hindering the formal, large-scale evaluations that have been slow to materialize [86].

The fact that the data are used for so many purposes is not just an issue for researchers, but for anyone using EHRs data not recorded by themselves [87]. Clinicians too must be aware that the patient information they share may not be complete, precise, or current. The same is true for health insurers, who rely on quality-of-care indicators derived from EHRs [44]. The LHS concept allows for greater attention to be paid to the context in which data are recorded in the EHR system, to develop mechanisms for decision support to prospectively address known “information gaps” and to track the provenance of data more thoroughly.

### Toward a Data Quality Research Agenda

In this paper, we have considered potential sources of bias in routinely available health data and mapped them onto the steps generally taken in the production and analysis of such data. For each step, we presented an overview of possible sources of bias that might lead to incomparable or invalid analysis results. We proposed a stepwise, purpose- and actor-oriented approach to understanding these factors and assessing their consequences. The size and direction of the effects from differences in health systems, of access to data by patients, of strategic recording behavior by health care professionals, of the absence or presence of recording guidelines and data quality interventions, and of different EHR systems are all largely unknown and present a huge risk to, potentially inflated, expectations of real-world data.

Unless data quality issues are better understood and unless adequate controls are embedded throughout the data lifecycle, data-driven health care will not live up to its expectations. Understanding these mechanisms is a multidisciplinary task, where medicine, health systems research, health services research, legal experts, and medical informatics have to reach out to each other and understand each other’s language.

For now, the factors mentioned summarized in Textbox 1 can be used as a checklist for anyone using or reusing EHR data. However, more targeted research is needed into the actual size of the possible sources of bias described in this paper. In the meantime, it is important for researchers, EHR vendors, and health policy makers to be aware that anything they do may have an effect on the quality of EHR data and the validity of outcomes from these data. We hope this paper will help to establish this awareness and provides input for a data quality research agenda. The possible sources of bias described in this paper can be used as hypotheses for this research agenda.

## Abbreviations

BP: blood pressure
CTSA: Clinical Translational Science Awards
DSSs: decision support systems
EHR: electronic health record
GP: general practitioner
LHS: learning health system
QOF: Quality and Outcomes Framework
TRANSFoRm: Translational Research and Patient Safety in Europe
